# Comprehensive Computer-Aided Decision Support Framework to Diagnose Tuberculosis From Chest X-Ray Images: Data Mining Study

**DOI:** 10.2196/21790

**Published:** 2020-12-07

**Authors:** Muhammad Owais, Muhammad Arsalan, Tahir Mahmood, Yu Hwan Kim, Kang Ryoung Park

**Affiliations:** 1 Division of Electronics and Electrical Engineering Dongguk University Seoul Republic of Korea

**Keywords:** tuberculosis, computer-aided diagnosis, chest radiograph, lung disease, neural network, classification-based retrieval

## Abstract

**Background:**

Tuberculosis (TB) is one of the most infectious diseases that can be fatal. Its early diagnosis and treatment can significantly reduce the mortality rate. In the literature, several computer-aided diagnosis (CAD) tools have been proposed for the efficient diagnosis of TB from chest radiograph (CXR) images. However, the majority of previous studies adopted conventional handcrafted feature-based algorithms. In addition, some recent CAD tools utilized the strength of deep learning methods to further enhance diagnostic performance. Nevertheless, all these existing methods can only classify a given CXR image into binary class (either TB positive or TB negative) without providing further descriptive information.

**Objective:**

The main objective of this study is to propose a comprehensive CAD framework for the effective diagnosis of TB by providing visual as well as descriptive information from the previous patients’ database.

**Methods:**

To accomplish our objective, first we propose a fusion-based deep classification network for the CAD decision that exhibits promising performance over the various state-of-the-art methods. Furthermore, a multilevel similarity measure algorithm is devised based on multiscale information fusion to retrieve the best-matched cases from the previous database.

**Results:**

The performance of the framework was evaluated based on 2 well-known CXR data sets made available by the US National Library of Medicine and the National Institutes of Health. Our classification model exhibited the best diagnostic performance (0.929, 0.937, 0.921, 0.928, and 0.965 for F1 score, average precision, average recall, accuracy, and area under the curve, respectively) and outperforms the performance of various state-of-the-art methods.

**Conclusions:**

This paper presents a comprehensive CAD framework to diagnose TB from CXR images by retrieving the relevant cases and their clinical observations from the previous patients’ database. These retrieval results assist the radiologist in making an effective diagnostic decision related to the current medical condition of a patient. Moreover, the retrieval results can facilitate the radiologists in subjectively validating the CAD decision.

## Introduction

According to a World Health Organization (WHO) report, tuberculosis (TB) is a major global health problem that causes severe medical conditions among millions of people annually. It ranks along with the HIV as a leading cause of mortality worldwide [1]. In 2014, approximately 9.6 million new TB cases were reported as per the WHO report, which ultimately caused 1.5 million deaths [1]. Today, early diagnosis and proper treatment can cure almost all the TB cases. Various types of laboratory tests have been developed to diagnose TB [2,3]. Among these tests, sputum smear microscopy is the most common, in which bacteria are examined from sputum samples using a microscope [2]. Developed in the last few years, molecular diagnostics [3] are the new techniques to diagnose TB. However, they may not be suitable in real-time screening applications. Currently, chest radiography is the most common test to detect pulmonary TB worldwide [4]. It has become cheaper and easier to use with the advent of digital chest radiography [5]. However, all these diagnostic tests are assessed by specialized radiologists, who must expend significant time and effort to make an accurate diagnostic decision. Therefore, such subjective methods may not be suitable for real-time screening.

Over the past few years, researchers have made a significant contribution to the development of computer-aided diagnosis (CAD) tools related to chest radiography [6,7]. Such automated tools can detect the various type of chest abnormalities within seconds and can aid in population screening applications, particularly in scenarios which lack medical expertise. Fortunately, the recent development in artificial intelligence has presented a remarkable breakthrough in the performance of these tools. Deep learning algorithms, specifically artificial neural networks [8], are the state-of-the-art achievement in the artificial intelligence domain. These algorithms offer more reliable methods to distinguish positive and negative TB cases from chest radiographs (CXR) images in a fully automated manner. In recent decades, several ground-breaking CAD methods have been proposed for TB diagnosis [9-24]. Most of the previous studies used segmentation-, detection-, and classification-based approaches to make the ultimate diagnostic decisions. All these methods indicated a binary decision (either TB positive or TB negative) without providing further descriptive information that may assist medical experts to validate the CAD decision. As the CAD decision can also be erroneous in some scenarios, a method to perform its cross-validation is necessary. Therefore, further research is required to achieve the practical performance and usability of such diagnostic systems in the real world. A comprehensive analysis of these existing studies [9-24] in comparison with our proposed method can be found in Multimedia Appendix 1.

Recently, various types of artificial neural networks have been proposed in the domain of general image processing to achieve the maximum performance in terms of accuracy (ACC) and computational cost. Among these models, convolutional neural networks (CNNs) [25] attract special attention because of their outstanding performance in many general and medical image recognition applications [26,27]. The entire structure of a CNN model consists of an input layer, hidden layers, and a final output layer. Among all these layers, hidden layers are considered the main components of the CNN model and primarily consist of a series of convolutional layers that include trainable filters of different sizes and depths. These filters are trained by performing a training procedure to extract the deep features from a training data set. When the training procedure is completed, the trained network can analyze the given testing data and generate the desired output.

In this paper, a novel CAD framework is proposed to diagnose TB from a given CXR image and provide the appropriate visual and descriptive information from a previous database, which can further assist radiologists to subjectively validate the computer decision. Thus, both subjective and CAD decisions will complement each other and ultimately result in effective diagnosis and treatment. The performance of our proposed framework was evaluated using 2 well-known CXR data sets [9,28]. The overall performance of our method is substantially higher than that of various state-of-the-art methods. The main contributions of our work can be summarized as follows:

1. To the best of our knowledge, this is the first comprehensive CAD framework in chest radiography based on multiscale information fusion that effectively diagnoses TB by providing visual and descriptive information based on a previous patients’ database.
2. We propose an ensemble classification model obtained by integrating 2 CNNs named shallow CNN (SCNN) to capture the low-level features such as edge information and a deep CNN (DCNN) to extract high-level features such as TB patterns.
3. Furthermore, a multilevel similarity measure (MLSM) algorithm is proposed based on multiscale information fusion to retrieve the best-matched cases from a previous database by computing a weighted structural similarity (SSIM) score of multilevel features.
4. The cross-data analysis (trained with one data set and tested with another data set, and vice versa) is a key measure to access the generalizability of a CAD tool. However, in the medical image analysis domain, most of the existing studies [9-15,18,19,21-24] did not analyze the performance of their methods in cross data set. Therefore, to further highlight the discriminative power of the proposed model in real-world scenarios, we also analyzed its performance in a cross data set.

The remainder of the paper is structured as follows. In the “Methods” section, we describe our proposed framework. Subsequently, the experimental results along with the data set, the experimental setup, and the performance evaluation metrics are provided in the “Results” section. Finally, the “Discussion” section presents the comprehensive discussions of our paper including the principal findings.

## Methods

This section presents a comprehensive description of our proposed framework in the following sequential order. First, we provide a brief overview of the proposed method to describe its end-to-end workflow. Subsequently, a detailed explanation of our proposed classification model and similarity measuring algorithm is presented in subsequent subsections.

### Overview of Our Proposed Framework

**Figure 1:**
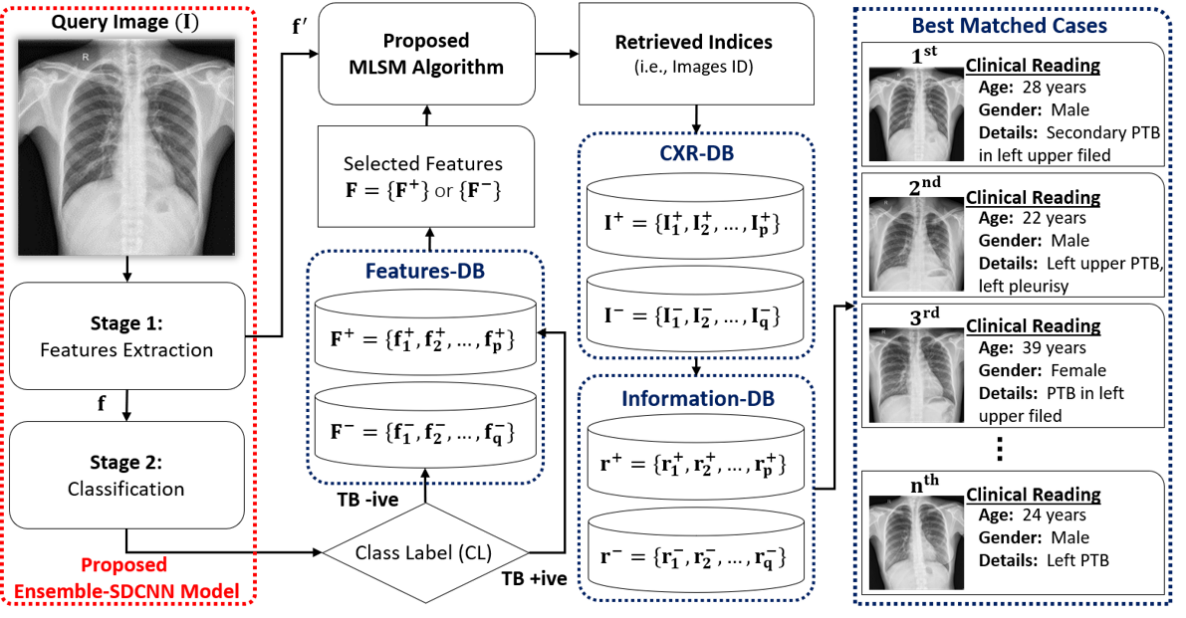
Comprehensive flow diagram of the proposed classification and retrieval framework. In the first stage, the given input CXR image is categorized as either TB positive or TB negative. In the second stage, the n best relevant cases are retrieved from the previous database based on our proposed MLSM algorithm. The parameter n is a user given input and controls the total number of retrieved cases from the previous record related to a current medical condition. CXR: chest radiograph; DB: database; MLSM: multilevel similarity measure; SDCNN: shallow–deepCNN; TB: tuberculosis.

In general, the overall performance of the image classification and retrieval framework is directly related to the mechanism of feature extraction, which is adopted to transform the visual data from high-level semantics to low-level features. These low-level features incorporate the distinctive information that can easily distinguish the instances of multiple classes. Recently, deep learning methods provide a fully automated means to extract the optimal features from available training data sets and lead to a substantial performance gain. In this study, we used the strengths of such deep learning methods to develop a comprehensive CAD tool to diagnose TB from CXR images. A comprehensive representation of the proposed framework is shown in Figure 1. The complete framework comprised a classification stage, a retrieval phase to perform the diagnostic decision, and retrieval of the descriptive evidence, respectively. In the first phase, our proposed ensemble-shallow–deep CNN (ensemble-SDCNN) model was trained to make the diagnostic decision for the given CXR image I by predicting its class label (CL) as either TB positive or TB negative. Such a diagnostic decision was made into 2 stages: feature extraction and classification. The detailed explanation of the proposed ensemble-SDCNN model and its workflow is provided in the subsequent subsection.

In the second phase, a classification-driven retrieval was performed for the input query image. The ultimate objective of this phase was to retrieve the relevant cases (such as CXR images) corresponding to the given CXR image with the inclusion of clinical observations (such as textual description) from the previous patients’ database. Such retrieval results can assist radiologists to subjectively validate the computer diagnostic decision, which ultimately results in an effective diagnostic decision. Initially, based on the predicted CL (in the first phase), a set of positive or negative feature vectors was selected from features database based on the following predefined criteria: F = F^+^, if CL = TB positive; otherwise F = F^–^, where F^+^and F^–^present the set of positive (F^+^ = {f_1_^+^, f_2_^+^, ..., f_p_^+^}) and negative features maps (F^–^ = {f_1_^–^, f_2_^–^, ..., f_q_^–^}) in the features database, respectively, and p and q are the total numbers of positive and negative cases, respectively.

Both F^+^ and F^–^ were extracted from TB-positive and TB-negative CXR-database (previously collected CXR images of different patients), respectively, and stored as a features database. In the subsequent step, our proposed MLSM algorithm was applied to select a subset of n best-matched features from this selected set of positive or negative features maps (ie, F={F^+^} or {F^–^}) in the first phase. Such feature matching was performed for the extracted multilevel features f′ of input query image I (as explained in a later subsection). Finally, the selected subset of n best-matched features was used to select the corresponding CXR images and their clinical readings from CXR-database and information database, respectively.

### Classification Network

**Figure 2:**
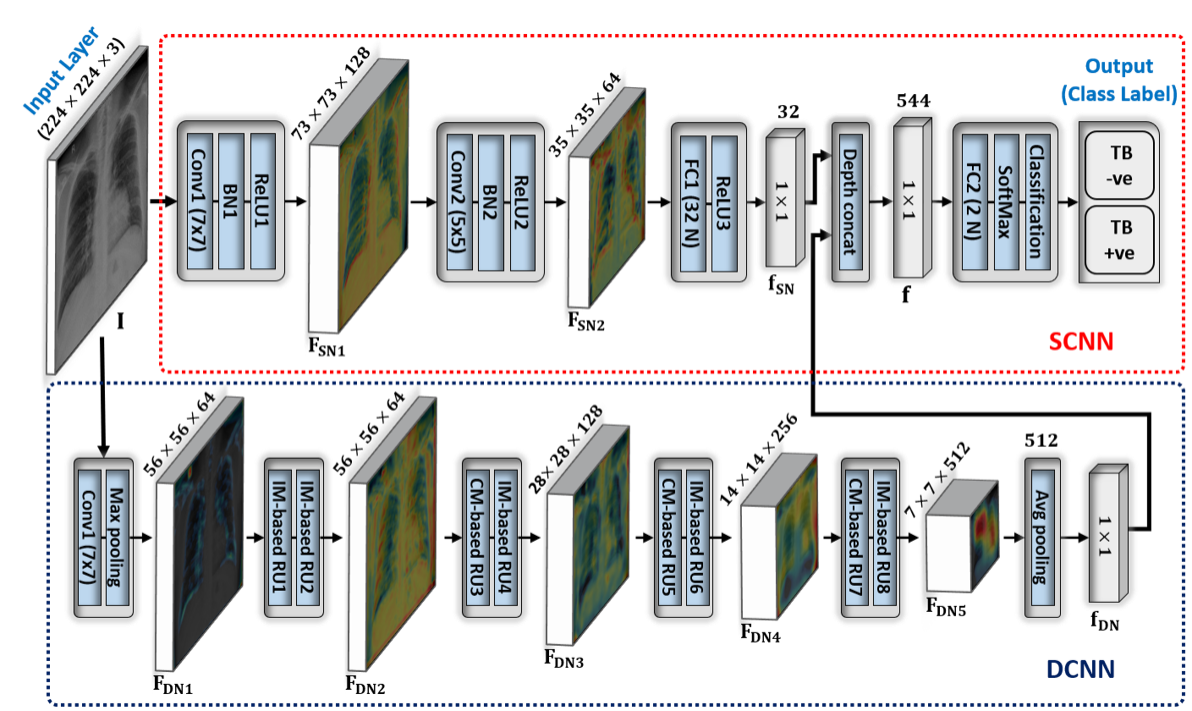
Overall architecture of our ensemble-SDCNN model by connecting 2 different networks, SCNN and DCNN. Both networks process the input image I simultaneously (in the testing phase) and extract 2 different feature vectors, which are concatenated and finally used to make a diagnostic decision by predicting the CL. CL: class label; CNN: convolutional neural network; DCNN: deep CNN; SCNN: shallow CNN; SDCNN: shallow–deep CNN.

The first phase of our proposed framework involved classifying the given CXR image as either TB positive or TB negative by predicting its CL. To accomplish this task, we proposed a jointly connected ensemble-SDCNN model by performing a features-level fusion of 2 different networks, SCNN and DCNN (Figure 2). In general, a shallow network captures low-level features such as edge information while a deep model is used to exploit high-level information such as overall shape patterns. In our radiograph image analysis study, the experimental results prove that the combination of low- and high-level features results in better performance compared with using only high-level features. Therefore, both networks were combined in parallel (by connecting their input and last output layers with each other; Figure 2) to create a single end-to-end trainable network. An existing DCNN model called a residual network (ResNet18) [29] was selected based on its substantial classification performance and the optimal number of parameters in comparison with the other CNN models. After selecting an optimal DCNN model, we further enhanced its performance by connecting our proposed SCNN model in parallel to it. Several experiments were performed to select the optimal number of convolutional and fully connected (FC) layers (and their hyper parameters) for the SCNN. The ultimate objective of these experiments was to construct an optimal shallow network (according to the number of parameters) that could maximize the overall classification performance of the complete network.

A complete layer-wise configuration of these models is shown in Table 1. This information can assist in exploring the parametric configuration of these models more precisely. Moreover, Figure 2 shows the overall architecture of the proposed ensemble-SDCNN model based on shallow and deep networks. Both SCNN and DCNN models processed the given CXR image in a parallel order to extract low- and high-level features, respectively. In the SCNN, the Conv1 layer (first convolutional layer with a total of 128 filters of size 7 × 7) explored the input image I in both horizontal and vertical directions and generated the output feature map, F_SN1_ of size 73 × 73 × 128. This output feature map was further processed through the Conv2 layer (second convolutional layer with a total of 64 filters of size 5 × 5) and converted into a new features map F_SN2_ of size 35 × 35 × 64. Thereafter, the FC1 layer (first fully connected layer including a total of 32 output nodes) identified the significant hidden patterns in F_SN2_ by combining all the learned features into a single features vector f_SN_ of size 1 × 1 × 32. Thus, we obtained a low-dimension features vector f_SN_that held a more diverse representation of the low-level features compared with F_SN2_.

**Table 1:** Layer-wise configuration details of the proposed ensemble-SDCNN^a^ model.^b^

Layer name		Output size^c^	Filter size^d^	Iterations	Parameters	
**DCNN^e^ model**						
	Input	(224,224,3)	N/A^f^	—^g^	—	
	Conv1	(112,112,64)	(7,7,64)	1	9600	
	Max pooling	(56,56,64)	(3,3)	1	—	
	IM^h^-based RU1^i^	(56,56,64)	(3,3,64)	2	74,112	
	IM-based RU2	(56,56,64)	(3,3,64)	2	74,112	
	CM^j^-based RU3	(28,28,128)	(3,3,128); (1,1,128)	2; 1	230,528	
	IM-based RU4	(28,28,128)	(3,3,128)	2	295,680	
	CM-based RU5	(14,14,256)	(3,3,256); (1,1,256)	2; 1	919,808	
	IM-based RU6	(14,14,256)	(3,3,256)	2	1,181,184	
	CM-based RU7	(7,7,512)	(3,3,512); (1,1,512)	2; 1	3,674,624	
	IM-based RU8	(7,7,512)	(3,3,512)	2	4,721,664	
	Avg pooling	(1,1,512)	(7,7)	1	—	
**SCNN^k^ model**						
	Conv1	(112,112,128)	(7,7,128)	1	19,200	
	Conv2	(35,35,64)	(5,5,64)	1	204,992	
	FC1	(1,1,32)	(5,5,64)	1	2,508,832	
	Depth concat	(1,1,544)	—	1	—	
	FC2	(1,1,2)	—	1	1090	
	SoftMax	(1,1,2)	—	1	—	
	Classification	2	—	1	—	

^a^SDCNN: shallow–deep CNN.

^b^Total learnable parameters: 13,915,426.

^c^Output size (image width, image height, # of channels),

^d^Kernel size (kernel width, kernel height, # of filters), Max pooling (kernel width, kernel height), Avg pooling (kernel width, kernel height).

^e^DCNN: deep CNN.

^f^N/A: not applicable.

^g^—: not available.

^h^IM: identity mapping.

^i^RU: residual unit.

^j^CM: convolutional mapping.

^k^SCNN: shallow CNN.

Similarly, for the DCNN, the input image I passes through a large number of convolutional layers (as compared with the SCNN) to exploit the high-level features. Our selected DCNN model was composed of multiple residual units (RUs) that consisted of identity mapping–based or convolutional mapping–based shortcut connections to each pair of 3 × 3 filters [29]. These shortcut connections caused the network to converge more efficiently compared with other sequential networks without including any shortcut connection. Moreover, a detailed explanation of these RUs is provided in [30]. Figure 2 also depicts an abstract representation of our selected DCNN model. Primarily, the input image I underwent the first convolutional layer, Conv1, with a total 64 filters of size 7 × 7. Subsequently, a Max pooling layer (with a window size 3 × 3) further down sampled the output of Conv1 and generated an intermediate features map F_DN1_ of size 56 × 56 × 64. Thereafter, a stack of 8 consecutive RUs (including 5 identity mapping–based RUs and 3 convolutional mapping–based RUs, as shown in Figure 2) further exploited high-level features. Furthermore, each RU converted the preceding features map into a new one by exploiting much deeper features in comparison with the previous layer. In Figure 2, all the intermediate features maps (ie, F_DN2_, F_DN3_, F_DN4_, and F_DN5_) after each pair of RU show the progressive effect of different RUs. We observed that the depth of these features maps increased progressively, and the spatial size decreased after passing through the RUs. Ultimately, a low-dimension feature vector, f_DN_, of size 1 × 1 × 512 was obtained after processing the final features map, F_DN5_ (obtained from the last RU), through an average pooling layer. This low-dimension feature vector exhibited a high-level abstraction of the input image I and substantially contributed, together with f_SN_, to the prediction of the final CL.

After extracting both low- and high-level features, a depth concatenation layer (labeled as Depth concat in Figure 2 and Table 1) performed the feature-level fusion by combining both f_SN_ and f_DN_ along the depth direction and generated a final features vector, f, of size 1 × 1 × 544. Finally, a stack of the FC2, SoftMax, and the classification layers (Figure 2) acted as a multilayer perceptron classifier and predicted the CL for the given image I using the ultimate features vector f. In this stack, the FC2 layer (including the number of nodes equal to the total number of classes) identified the larger patterns in fby combining all the features values. It multiplied f by a weight matrix W, and then added a bias vector b, where y = W·f + b, with y = [y_i_|_i=1,2_]. Subsequently, the SoftMax layer converted the output of FC2 in terms of probability by applying the softmax function as y′_i_=e^yi^/Σ^2^_i=1_ [8]. Ultimately, the classification layer obtained (y′_i_)from the SoftMax layer was assigned each input to one of the 2 mutually exclusive classes (ie, TB positive and TB negative) using a cross-entropy (CE) loss function as Loss_CE_(W,b) = Σ^2^_i=1_ c_i_ln(y′_i_) [8]. Here, (W, b) are the network trainable parameters and c_i_ is the indicator of the actual class label of the *i*th class during the training procedure. Meanwhile, in the testing phase, the network generated a single CL (as either TB positive or TB negative) corresponding to each input image I.

**Figure 3:**
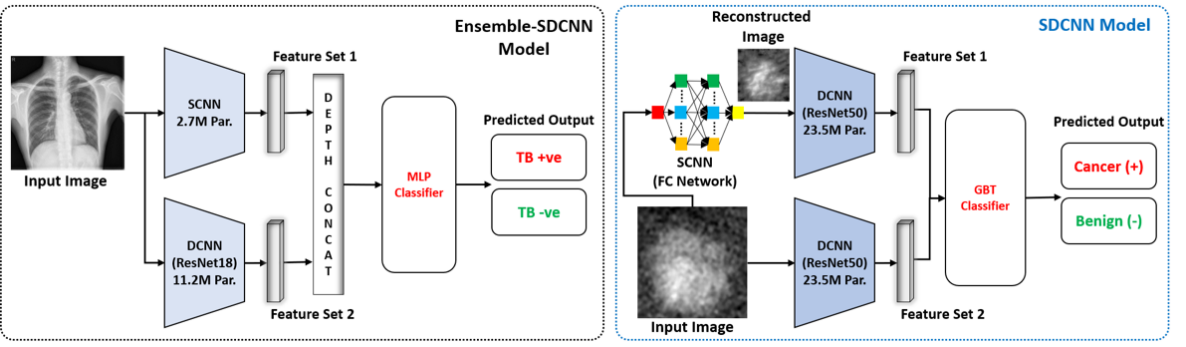
Overall structural comparison of our proposed ensemble-SDCNN (left) and existing SDCNN model (right). MLP: multilayer perceptron; GBT: gradient boosted tree.

There is also an existing SDCNN model [31] (proposed for effective breast cancer diagnosis). However, there is a substantial difference between our proposed and the existing model [31] in terms of architecture, application, and computational complexity. In [31], the authors proposed an ensemble of 2 existing ResNet50 [29] models to extract the deep features and then used a gradient boosted tree classifier to make the diagnostic decision. In addition, a 4-layer FC network, namely SCNN (which includes FC convolutional layers), was proposed for image reconstruction to increase the data samples in the preprocessing stage. By contrast, in our work, we proposed an ensemble of SCNN (which includes 2 convolutional layers [no FC] and 1 FC layer) and DCNN models as shown in Figure 2 to extract low- and high-level features, respectively. Then, an FC classifier (also known as a multilayer perceptron) was used to make the final diagnostic decision using both low- and high-level features. Furthermore, the SCNN [31] is an image reconstruction network (ie, both input and output are images), whereas our proposed SCNN is a classification network (ie, input is image, and output is feature vector). Therefore, the architecture of both SCNN models is completely different from each other. In addition, our DCNN model is based on ResNet18 that includes a substantially lower number of trainable parameters than ResNet50 as used in [31], that is, 11.2M (ResNet18) << 23.5M (ResNet50). In this way, the total number of trainable parameters of the proposed ensemble-SDCNN is substantially lower than the existing SDCNN [31], that is, 13.9M (proposed) << 47M [31]. Figure 3 further highlights the overall structural difference between our proposed and the existing model [31].

### Multilevel Similarity Measure Algorithm

In the medical domain, the visually correlated images occasionally depict different illnesses, whereas the images for a similar ailment have distinctive appearances. Therefore, estimating the similarity by contemplating the multilevel features is more advantageous in content-based medical image retrieval systems rather than using single-level features. Most of the existing systems often use a single-level similarity measure (SLSM) method to perform the content-based medical image retrieval task. However, it can miss the potentially useful information that is required in discriminating the different diseases in visually correlated images. To overcome these challenges, we proposed an MLSM algorithm to retrieve the best-matched cases from the previous patients’ database by fusing multilevel features starting from a low-level visual to a high-level semantic scale. The similarity at multiple features levels was calculated using a well-known matching algorithm called SSIM [32], as it quantified the visibility of errors (differences) between 2 samples more appropriately compared with other simple matching schemes such as mean square error, peak signal-to-noise ratio (PSNR), and Euclidean distance. A generalized mathematical expression to calculate the SSIM score between 2 samples (x and y) is given as follows:

SSIM(x,y) = ([2µ_x_µ_x_ + c1][2σ_xy_ + c_2_])/[µ^2^_x_ + µ^2^_y_ + c_1_] [σ^2^_x_ + σ^2^_y_ + c_2_]     **(1)**

where [µ_x_·µ_y_], [σ_x_·σ_y_], and σ_xy_ are the local mean, standard deviation, and cross-covariance of the given samples, respectively; and c_1_ and c_2_ are constants to avoid instabilities such as infinity errors and undefined solutions.

**Figure 4:**
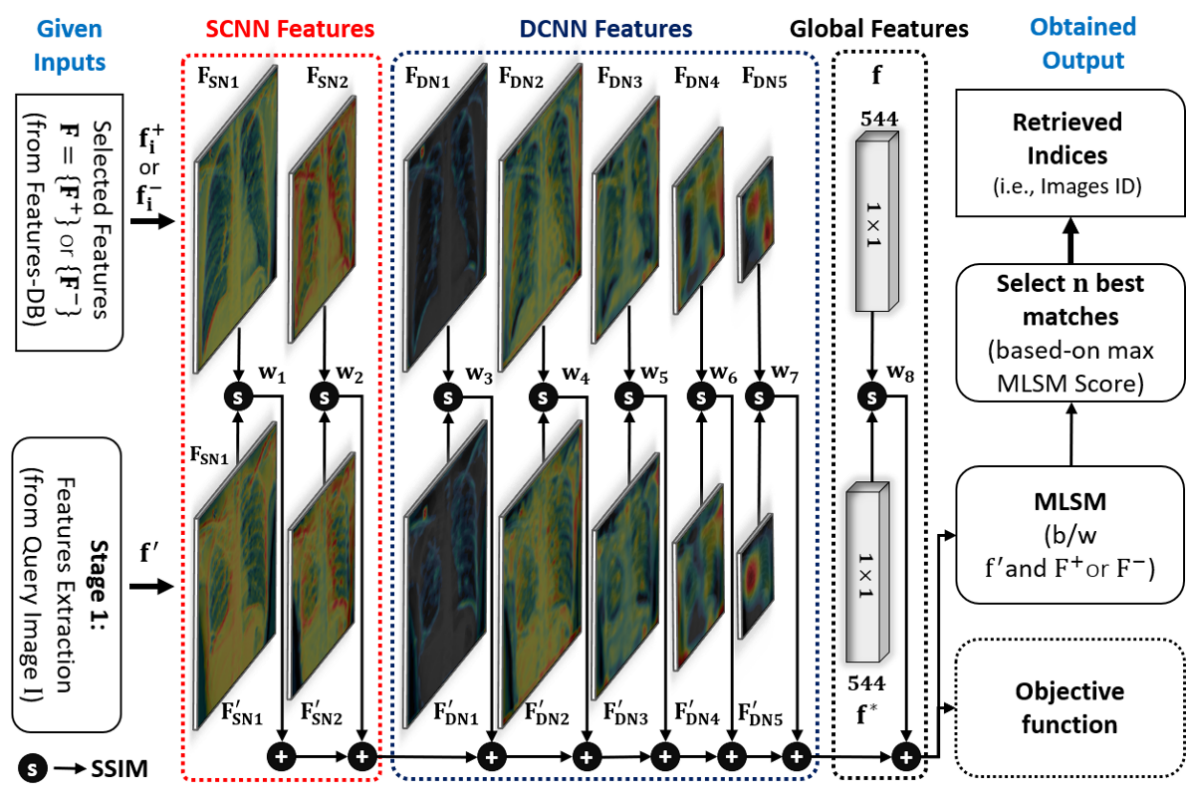
Complete workflow diagram of our proposed MLSM algorithm using the multilevel features (extracted from the different parts of the proposed ensemble-SDCNN model) in retrieving the best-matched cases from a previous patients’ database. DCNN: deep convolutional neural network; MLSM: multilevel similarity measure; SCNN: shallow convolutional neural network; SSIM: structure similarity.

In our MLSM algorithm, multilevel features were extracted from the 8 different locations of the ensemble-SDCNN model (Figure 4). Each features map in Figure 4 was obtained by calculating the depth-wise averaging of each stack of feature maps (extracted from a particular location). Moreover, this newly obtained feature map corresponding to each specific location was further presented with a pseudocolor scheme to highlight the activated regions more appropriately. In Figure 4, f′ presents a set of these multilevel features maps (ie, {F′_SN1_, F′_SN2_, F′_DN1_, F′_DN2_, F′_DN3_, F′_DN4_, F′_DN5_, f*}) corresponding to the given query image I. Similarly, f^+^_i_ or f^–^_i_ notates a set of multilevel features maps (ie, {F_SN1_, F_SN2_, F_DN1_, F_DN2_, F_DN3_, F_DN4_, F_DN5_, f}) for the *i*th positive or negative sample image in CXR-database, respectively. The selection of f^+^_i_ or f^–^_i_ was conducted based on the CL prediction, which was performed by our proposed network in the first phase. For example, in a positive prediction (ie, CL = TB positive) for the input query image I, the MLSM score between the query image I and set of p positive sample images I^+^ (stored in CXR-database) is calculated as follows:

MLSM = Σ^8^_k=1_w_k_SSIM(f′{k},f^+^_i_{k}) _i=1, 2, …, p_     **(2)**

Similarly, in a negative prediction (ie, CL = TB negative), the MLSM score between the query image I and set of q negative sample images I^–^ (also stored in CXR-database) is calculated as follows:

MLSM = Σ^8^_k=1_w_k_SSIM(f′{k},f^–^_i_{k}) _i=1, 2, …, q_     **(3)**

In both mathematical expressions, w_1_, w_2_, w_3_, …, w_8_ are the weights of SSIM measured at different levels and their total sum is equal to one (ie, Σ^8^_i=1_ w_i_=1). The optimal weights were obtained by maximizing the intraclass SSIM score for some selected pairs of positive CXR images. Each pair (I^+^_i_, I^+^_j_) was selected from the positive data samples based on the highly correlated clinical observations between 2 CXR images. These observations were provided in our selected data sets as a text file for each data sample. As our main objective was to diagnose TB by retrieving similar abnormal cases from a previous database, we only considered positive CXR images in calculating the optimal weights rather than using normal images.

Finally, the overall objective function to maximize the intraclass similarity is defined as follows:

w*=max(Σ_i,j_εTBpositiveΣ^8^_k=1_w_k_ SSIM[f^+^_i_{k},f^+^_j_{k}]) /N^+^     **(4)**

where N^+^is the total number of pair images selected from the positive data samples. In our experiment, the total number of pairs was 30 (ie, N^+^ = 30). After performing the optimization according to Equation (4), we obtained the optimal values of w_1_, w_2_, w_3_, …, w_8_ as 0.069, 0.179, 0.087, 0.133, 0.071, 0.123, 0.299, and 0.039, respectively. Finally, these optimal weights were used to calculate the MLSM scores between f′ and F^+^ (set of positive features maps in features database) or F^–^ (set of negative features maps in features database) depending on the predicted CL in the classification stage. Thereafter, the indices of n best-matched features were selected based on the maximum MLSM scores. These indices were eventually used to select the corresponding CXR images and their clinical readings from CXR-database and information database, respectively. Thus, n best-matched cases were retrieved from the previous patients’ database, which could assist radiologists in making an effective diagnostic decision after performing the subjective validation of the computer decision.

## Results

### Data Set and Preprocessing

Our proposed diagnostic framework was validated using 2 publicly available data sets: Montgomery County (MC) and Shenzhen (SZ) [9,28]. The MC data set is a subset of a larger CXR repository collected within the TB control program of the Department of Health and Human Services of Montgomery County, Maryland, USA. All these images are in 12-bit grayscale, captured using a Eureka stationary X-ray machine. This data set comprises a total of 138 posteroanterior CXR images, among which there are 80 normal and 58 abnormal images with the manifestations of TB disease. The abnormal images encompass a vast range of abnormalities related to pulmonary TB. The SZ data set is collected from the Shenzhen No. 3 People’s Hospital in Shenzhen, Guangdong Providence, China. This data set includes a total of 326 normal and 336 abnormal CXR images, which include different types of abnormalities related to pulmonary TB. All these images are also in 12-bit grayscale and were captured using the Philips DR DigitalDiagnost system. In both data sets, a radiologist report is also provided for each CXR image as a text file, containing the clinical observation related to chest abnormalities along with the patient’s age and gender information. After collecting both data sets, we resized all the images to a spatial dimension of 224 × 224 (according to the fixed input layer size of our ensemble-SDCNN model).

### Implementation Details

The proposed framework was implemented using a standard deep learning toolbox available in the MATLAB R2019a (MathWorks, Inc.) framework [33]. It provides a complete framework for developing and testing different types of artificial neural networks and using existing pretrained networks. All the experiments were performed on a desktop computer with a 3.50-GHz Intel Core i7-3770K CPU [34], 16-GB RAM, an NVIDIA GeForce GTX 1070 graphics card [35], and Windows 10 operating system (Microsoft). Our proposed and other baseline models were trained through back-propagation (a procedure to determine the optimal parameters of a model) using a well-known optimization algorithm called the stochastic gradient descent [36]. It iteratively trains the network by computing the optimal learnable parameters (such as filter weights and biases) that are included in different layers of the network. The following hyper-parameters were selected for our proposed and all the comparative CNN-based methods: learning rate as 0.001 with a drop factor of 0.1. Moreover, the min-batch size was selected as 10 (ie, feeding a stack of 10 images per gradient update in each iteration), L2-regularization as 0.0001, and a momentum factor as 0.9.

### Evaluation Metrics and Protocol

After the training, the quantitative performance of our proposed framework was evaluated based on the following metrics: ACC, average precision (AP), average recall (AR), F1 score (F1), and finally the area under the curve (AUC) [37]. These well-known metrics can quantify the overall performance of a deep learning model from many perspectives. The mathematical definition of all these metrics is provided in Table 2.

In our binary classification problem, true positive (TP) and true negative (TN) were the outcomes of our model for correctly predicted positive and negative cases, respectively, whereas false positive (FP) and false negative (FN) could be interpreted as the incorrectly predicted positive and negative cases, respectively. Finally, these 4 different outcomes were further used in assessing the overall performance of a model in terms of ACC, AP, AR, F1, and AUC. We performed a fivefold cross-validation in all the experiments by randomly selecting 80% of data (110/138 [79.7%] of MC data and 530/662 [80.0%] SZ data) for training and the remaining 20% (28/138 [20.2%] of MC data and 132/662 [19.9%] SZ data) for testing. As most of the previous studies considered fivefold cross-validations, we followed a similar data splitting protocol. However, the fivefold cross-validation was not possible for the evaluation of the cross–data set performance, as a complete data set was used for training and others for testing. However, we performed cross-data validation using the MC data set as training and the SZ data set as testing, and vice versa.

**Table 2:** Mathematical definition of our selected performance evaluation metrics.

Metric name	Mathematical equation
Accuracy (ACC)	(TP^a^ + TN^b^)/(TP + TN + FP^c^ + FN^d^)
Average precision (AP)	TP/(TP + FP)
Average recall (AR)	TP/(TP + FN)
F1 score (F1)	2×([AP × AR]/[AP + AR])
Area under the curve (AUC)	0.5 × (TP/[TP + FP] + TN/[TN + FP])

^a^TP: true positive.

^b^TN: true negative.

^c^FP: false positive.

^d^FN: false negative.

### Our Results and an Ablation Study

The overall performance of our diagnostic framework was directly related to the classification performance of the proposed ensemble-SDCNN model. As in our classification-driven retrieval framework, the first step was to predict the CL for the given query image and then explore that predicted class database to retrieve the relevant cases. Consequently, the correct prediction would ultimately result in correct retrieval and the incorrect prediction in incorrect retrieval. Therefore, we comprehensively assessed the classification performance of the proposed model for both data sets and their combinations. Table 3 shows the performance of our classification model along with an ablation study to highlight the significance of each submodel in enhancing the overall performance. Therefore, the individual performance of both SCNN and DCNN models was also computed as an ablation study. The experimental results indicated that the combination of SCNN and DCNN resulted in a substantial performance gain (ie, 8.8%, 8.12%, 9.42%, 8.76%, and 5.68% for the average F1, AP, AR, ACC, and AUC, respectively) compared with their individual performances. We further performed a *t* test [38] and Cohen *d* [39] analysis to signify the performance gain of our SDCNN model in contrast to the DCNN (second-best model). In these 2 performance analysis measures, a large number of experimental results appropriately discriminated the performances of 2 systems.

Therefore, the detailed performance results of both ensemble-SDCNN and DCNN for all the different folds were used to perform the *t* test and Cohen *d* analysis. In the *t* test analysis, all the *P*-values (ie, .012, .011, .015, .014, and .012 in the case of average F1, AP, AR, ACC, and AUC, respectively) were less than .05. These results implied the discriminative performance of our ensemble-SDCNN against the SCNN with a 95% confidence score. In the Cohen *d* analysis, the performance difference between 2 systems was measured in terms of effect size [40], which is generally categorized as small (approximately 0.2-0.3), medium (approximately 0.5), and large (≥0.8). The large effect size indicated a substantial performance difference between the 2 systems. In this analysis, all the effect sizes (ie, 0.6, 0.6, 0.6, 0.5, and 0.5 for the average F1, AP, AR, ACC, and AUC, respectively) were greater than and equal to 0.5, which also indicated the substantial performance difference between the ensemble-SDCNN and SCNN models.

**Table 3:** Classification performance of our proposed ensemble-SDCNN^a^ model including the submodels as an ablation study.

Data sets and models		F1	AP^b^	AR^c^	ACC^d^	AUC^e^
**MC^f^**						
	SCNN^g,h^	0.765	0.775	0.757	0.769	0.817
	DCNN^i,j^	0.88	0.888	0.872	0.878	0.932
	ensemble-SDCNN	0.929	0.937	0.921	0.928	0.965
**SZ^k^**						
	SCNN	0.802	0.803	0.802	0.802	0.868
	DCNN	0.892	0.892	0.892	0.891	0.939
	ensemble-SDCNN	0.908	0.909	0.908	0.908	0.948
**MC + SZ**						
	SCNN	0.79	0.793	0.788	0.789	0.841
	DCNN	0.891	0.892	0.89	0.89	0.943
	ensemble-SDCNN	0.9	0.902	0.898	0.899	0.95
**MC train and SZ test**						
	SCNN	0.557	0.559	0.555	0.557	0.541
	DCNN	0.54	0.574	0.51	0.517	0.737
	ensemble-SDCNN	0.795	0.798	0.793	0.792	0.853
**SZ train and MC test**						
	SCNN	0.625	0.624	0.626	0.616	0.601
	DCNN	0.7	0.702	0.698	0.71	0.754
	ensemble-SDCNN	0.811	0.808	0.813	0.797	0.873

^a^SDCNN: shallow–deep CNN.

^b^AP: average precision.

^c^AR: average recall.

^d^ACC: accuracy.

^e^AUC: area under the curve.

^f^MC: Montgomery County.

^g^Ablation study performance by only considering SCNN for classification.

^h^SCNN: shallow CNN.

^i^Ablation study performance by only considering DCNN for classification.

^j^DCNN: deep CNN.

^k^SZ: Shenzhen.

**Figure 5:**
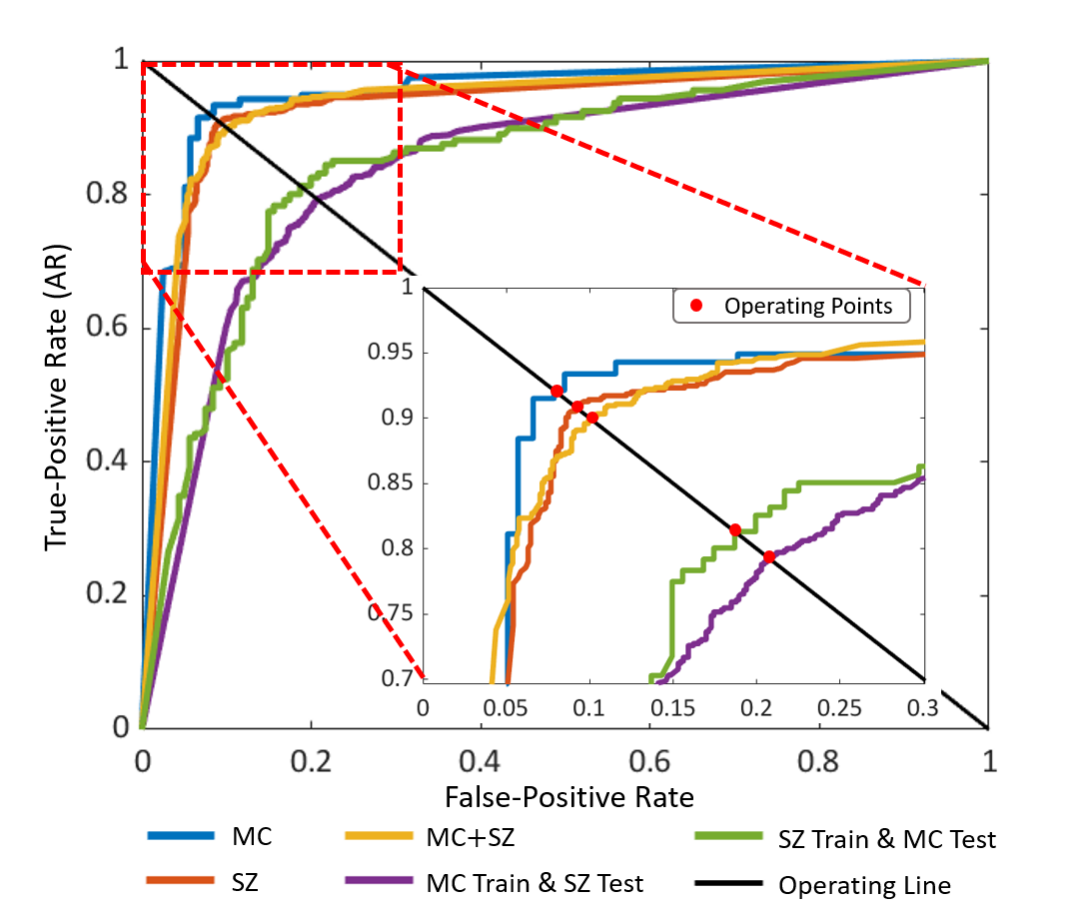
Receiver operating characteristic curves of our ensemble-SDCNN model for all the datasets. Each curve plots true-positive rate (TPR) vs false-positive rate (FPR) of our model at different classification thresholds beginning from 0 to 1 in 0.001 increments. MC: Montgomery County; SDCNN: shallow–deep convolutional neural network; SZ: Shenzhen.

Figure 5 depicts the receiver operating characteristic curves of the proposed model for all the data sets. Each curve plots the TPR versus the FPR of our model at different classification thresholds beginning from 0 to 1 at 0.001 increments. Among all the classification thresholds, the optimal threshold was obtained based on the operating points (as highlighted with red closed circles) existing over the operating line. We attained the optimal threshold value of 0.507 for all the data sets. This implied that any CXR image with a classification probability larger than .507 was reported as a positive case. Finally, based on these receiver operating characteristic curves, we calculated the AUC results of our model for each data set (Table 3). We observed that the MC, SZ, and MC + SZ data sets had comparable AUCs of 0.965, 0.948, and 0.95, respectively. However, the performance of the cross–data set AUC was lower than that of the MC and SZ because of high intraclass and interclass variances between 2 different data sets, but the comparative performance (as reported in the subsequent section) of our model was still greater than the existing state-of-the-art methods for all the data sets.

**Figure 6:**
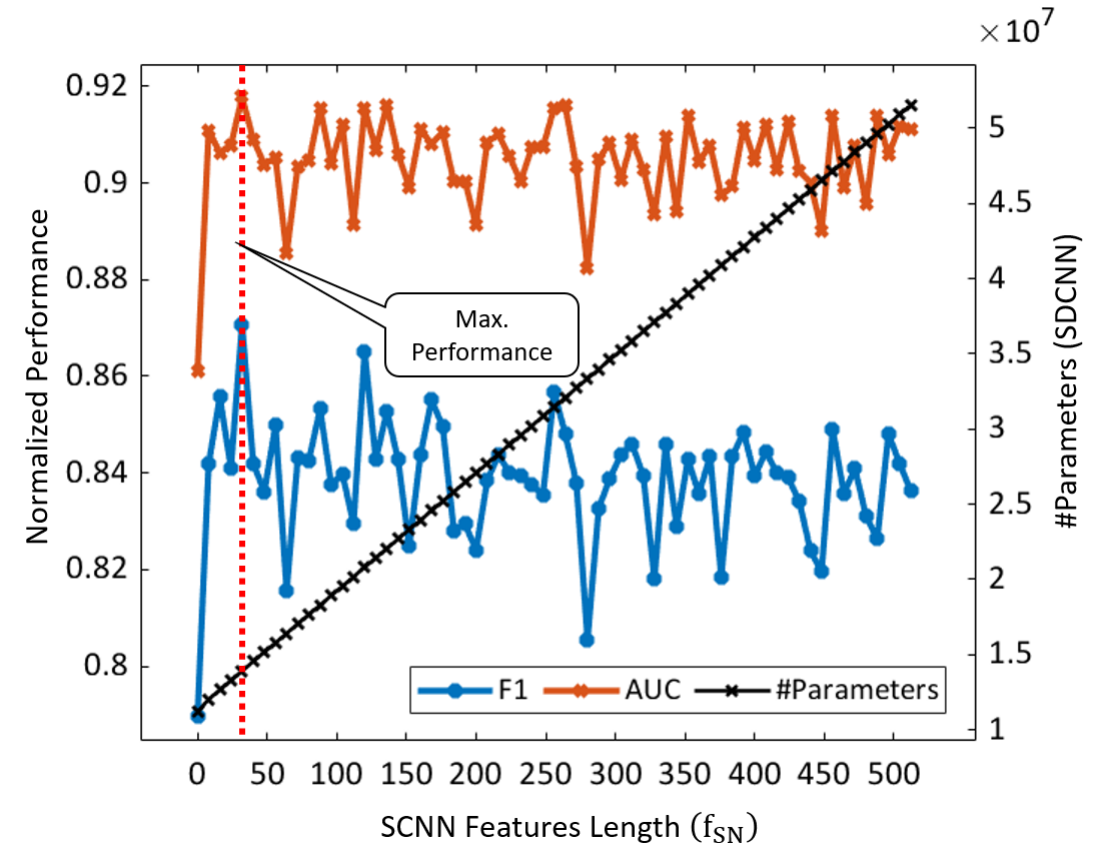
Average performance of the proposed ensemble-SDCNN model by considering different lengths of SCNN features with DCNN features (beginning from 0 to 512 with the increment of eight features in each experiment). AUC: area under the curve; DCNN: deep convolutional neural network; SDCNN: shallow–deep convolutional neural network; SCNN: shallow convolutional neural network.

To determine the optimal ratio of the SCNN features with the DCNN, we performed several experiments for all the data sets by considering the different feature lengths of f_SN_ concatenated with f_DN_. In this analysis, the feature lengths began from 0 to 512 with the increment of 8 features per experiment. Figure 6 shows the F1 and AUC results (average performance of all the data sets) according to different features length of f_SN_. In addition, the black line depicts the growing number of the total parameters of our proposed model with the increasing length of f_SN_. The figure indicates that our model exhibited the best performance (ie, maximum F1 of 0.871 and AUC of 0.918 as indicated by the vertical red line) and required the optimal number of total parameters as 1.39 × 10^7^ for f_SN_=32. Although the total number of trainable parameters of our model was slightly higher (approximately 2.7 million) than that of the DCNN, a substantial performance difference was observed, particularly for the cross data set (Table 3).

In our classification-driven framework, both classification and retrieval performances were similar. However, we also evaluated the retrieval performance without performing the class prediction to validate the superiority of our classification-driven approach. In Table 4, the experimental results indicate that our classification-driven approach exhibited higher retrieval accuracies than the retrieval without class prediction. Moreover, our retrieval approach was computationally more efficient than that without class prediction as feature matching was performed using only the predicted class database rather than the entire database as in the retrieval without class prediction. In conclusion, these comparative results (Tables 3 and 4) implied that our jointly connected model exhibited superior performance in making the effective diagnostic decision and retrieving the best-matched cases from the previous database.

**Table 4:** Comparative retrieval performance with and without predicting the class label (CL).

Retrieval and data sets		F1	AP^a^	AR^b^	ACC^c^
**Without class prediction**					
	MC^d^	0.844	0.861	0.828	0.847
	SZ^e^	0.891	0.892	0.89	0.89
	MC + SZ	0.88	0.882	0.878	0.879
	MC train and SZ test	0.534	0.538	0.53	0.533
	SZ train and MC test	0.729	0.737	0.72	0.739
**With class prediction**					
	MC	0.929	0.937	0.921	0.928
	SZ	0.908	0.909	0.908	0.908
	MC + SZ	0.9	0.902	0.898	0.899
	MC train and SZ test	0.795	0.798	0.793	0.792
	SZ train and MC test	0.811	0.808	0.813	0.797

^a^AP: average precision.

^b^AR: average recall.

^c^ACC: accuracy.

^d^MC: Montgomery County.

^e^SZ: Shenzhen.

### Comparative Analysis

Several CAD methods are presented in the literature for diagnosing pulmonary TB in CXR images. To make a fair comparison, we considered the following state-of-the-art methods [14,15,17,21,22,41,42], because these approaches selected the same data sets and experimental protocols as considered in our study. Moreover, in some recent studies [21], the authors adopted existing CNN models to classify the different types of pulmonary abnormalities including TB. However, these studies considered different data sets and experimental protocols. For a fair and detailed comparison, we evaluated the performance of these methods for our selected data sets and experimental protocol. Additionally, we calculated the performance of other CNN models [29,43-45] proposed for the general image-classification domain rather than radiology. The objective of this comparative analysis was to estimate the performance of the existing state-of-the-art CNN models in CXR image analyses. All these comparative analysis results are shown in Table 5.

**Table 5:** Comparative performance analysis of the proposed ensemble-SDCNN^a^ model with various state-of-the-art methods.

Comparative methods	MC^b^					SZ^c^							MC + SZ				
	F1	AP^d^	AR^e^	ACC^f^	AUC^g^		F1	AP	AR	ACC	AUC	F1		AP	AR	ACC	AUC
LBP^h^ and SVM^i,j^ [46]	0.537	0.58	0.5	0.58	0.675		0.76	0.76	0.76	0.76	0.83	0.729		0.729	0.729	0.729	0.763
HoG^k^ and SVM^i^ [47]	0.797	0.796	0.798	0.797	0.863		0.85	0.85	0.85	0.85	0.90	0.822		0.823	0.821	0.821	0.882
ShuffleNet^i^ [43]	0.747	0.771	0.727	0.748	0.84		0.875	0.876	0.873	0.873	0.937	0.884		0.885	0.883	0.884	0.936
InceptionV3^i^ [44]	0.739	0.773	0.711	0.74	0.828		0.882	0.883	0.881	0.881	0.942	0.887		0.89	0.884	0.885	0.944
MobileNetV2^i^ [45]	0.762	0.769	0.755	0.769	0.833		0.876	0.878	0.875	0.875	0.941	0.886		0.888	0.883	0.884	0.946
Santosh et al [41]	—^l^	—	—	0.79	0.88		—	—	—	0.86	0.93	—		—	—	—	—
Hwang et al [17]	—	—	—	0.674	0.884		—	—	—	0.837	0.926	—		—	—	—	—
ResNet50^i^ [29]	0.788	0.796	0.78	0.79	0.886		0.877	0.877	0.877	0.876	0.94	0.88		0.881	0.878	0.879	0.921
ResNet101^i^ [29]	0.8	0.821	0.782	0.798	0.895		0.864	0.865	0.862	0.861	0.934	0.859		0.862	0.857	0.858	0.923
Alfadhli et al [14]	—	0.81	0.79	0.791	0.89		—	—	—	—	—	—		—	—	—	—
GoogLeNet^i^ [20,21]	0.834	0.851	0.818	0.834	0.902		0.852	0.853	0.851	0.851	0.921	0.843		0.846	0.84	0.84	0.914
Lopes and Valiati [21]	—	—	—	0.826	0.926		—	—	—	0.847	0.904	—		—	—	—	—
Vajda et al [42]	—	—	—	0.783	0.87		—	—	—	—	—	—		—	—	—	—
Pasa et al [22]	—	—	—	0.79	0.811		—	—	—	0.844	0.9	—		—	—	0.862	0.925
Govindarajan and Swaminathan [15]	0.876	—	0.877	0.878	0.94		—	—	—	—	—	—		—	—	—	—
Proposed	0.929	0.937	0.921	0.928	0.965		0.908	0.909	0.908	0.908	0.948	0.9		0.902	0.898	0.899	0.95

^a^SDCNN: shallow–deep CNN.

^b^MC: Montgomery County.

^c^SZ: Shenzhen.

^d^AP: average precision.

^e^AR: average recall.

^f^ACC: accuracy.

^g^AUC: area under the curve.

^h^LBP: local binary pattern.

^i^We evaluated the performance of these models using our selected data sets and experimental protocol.

^j^SVM: support vector machine.

^k^HoG: histogram of oriented gradients.

^l^—: not available. These results were not reported in some existing studies.

We observed that our method exhibited a superior performance (in terms of all the performance measures and data sets) compared with all the other baseline methods. In addition to deep learning–based methods, we evaluated and compared the performance of 2 known handcrafted feature-based methods [46,47]. To evaluate the performance of these 2 methods [46,47], we used the following default parameters as provided by the MATLAB framework [33]: size of histogram of oriented gradients cell as 8 × 8 with block size of 2 × 2 and number of overlapping cells between adjacent blocks as 1 block and the number of orientation bins as 9. In local binary patterns (LBPs) [46], the number of neighbor pixels considered was 8, with the linear interpolation method applied to compute pixel neighbors. Whereas in LBP histogram parameters, cell size was selected as 1 × 1 by applying L2-normalization to each LBP cell histogram. Thus, our comparative analysis was more detailed than the various existing studies [14,17,21,22]. For the MC data set, the performance gain of our model in contrast to Govindarajan and Swaminathan [15] (second-best) was greater than 4.4%, 5%, and 2.5% for AR, ACC, and AUC, respectively. Similarly, the difference in the performance of our model from a second-best model called InceptionV3 [44] (for the SZ data set) was more than 2.6%, 2.6%, 2.7%, 2.7%, and 0.6% for F1, AP, AR, ACC, and AUC, respectively. Moreover, for the combined data set (MC + SZ), the performance gain of our model in contrast to InceptionV3 [44] (second-best) was equal to 2.1%, 1.9%, 2.4%, 2.3%, and 0.4% for F1, AP, AR, ACC, and AUC, respectively. Hence, the performance of all these existing baseline methods validated the superiority of our proposed model with a substantial performance difference.

Moreover, comparative studies on the analysis of the cross–data set performance are rare. The majority of the studies only considered a similar data set for training and testing. Cross–data set testing is an important analysis to demonstrate the general capability of a model and its potential applicability in a real-world environment. Therefore, similar comparative results are also evaluated (in a cross data set) for different baseline models for a detailed performance comparison with the proposed ensemble-SDCNN model. In this analysis, the MC data set was used to train the model and SZ was used to test, and vice versa. Table 6 shows the results of these cross–data set analyses along with comparative studies.

These comparative results indicated that our model had outperformed the various deep learning and handcrafted feature-based TB diagnostic methods. For the SZ data set, which was used for training, the accuracies were slightly higher than those for the MC data set. The main reason for this was the presence of more training data samples compared with the MC data set. For the scenario in which the MC data set was the training set and the SZ the testing set, the performance of our model in contrast to that of Santosh and Antani [16] (second best) was higher than 3.3%, 3.2%, and 3.3% for AR, ACC, and AUC, respectively, and the comparative performance difference of our model with that of Santosh and Antani [16] (for SZ as training and MC as testing data sets) was also higher than 2.3%, 1.7%, and 2.3% for AR, ACC, and AUC, respectively. All these experimental results highlighted the potential applicability of our model in real-world diagnostics related to chest abnormalities.

**Table 6:** Results of comparative performance analysis of our proposed method with various baseline methods for cross data sets.

Data sets and our methods		F1	AP^a^	AR^b^	ACC^c^	AUC^d^
**MC^e^ train and SZ^f^ test**						
	LBP^g^ and SVM^h,i^ [46]	0.496	0.492	0.5	0.492	0.69
	HoG^j^ and SVM^i^ [47]	0.664	0.695	0.635	0.639	0.762
	ShuffleNet^i^ [43]	0.661	0.715	0.615	0.61	0.709
	InceptionV3^i^ [44]	0.708	0.717	0.7	0.698	0.761
	MobileNetV2^i^ [45]	0.613	0.678	0.559	0.565	0.78
	ResNet50^i^ [29]	0.686	0.707	0.667	0.663	0.77
	ResNet101^i^ [29]	0.674	0.677	0.671	0.672	0.772
	GoogLeNet^i^ [20,21]	0.592	0.595	0.589	0.591	0.65
	Santosh and Antani [16]	—^k^	—	0.76	0.76	0.82
	Proposed	0.795	0.798	0.793	0.792	0.853
**SZ train and MC test**						
	LBP and SVM^i^ [46]	0.537	0.58	0.5	0.58	0.552
	HoG and SVM^i^ [47]	0.559	0.573	0.546	0.594	0.601
	ShuffleNet^i^ [43]	0.633	0.643	0.624	0.652	0.683
	InceptionV3^i^ [44]	0.681	0.722	0.644	0.688	0.748
	MobileNetV2^i^ [45]	0.668	0.772	0.589	0.652	0.797
	ResNet50^i^ [29]	0.64	0.642	0.638	0.616	0.787
	ResNet101^i^ [29]	0.641	0.726	0.574	0.638	0.698
	GoogLeNet^i^ [20,21]	0.648	0.691	0.609	0.659	0.754
	Santosh and Antani [16]	—	—	0.79	0.78	0.85
	Proposed	0.811	0.808	0.813	0.797	0.873

^a^AP: average precision.

^b^AR: average recall.

^c^ACC: accuracy.

^d^AUC: area under the curve.

^e^MC: Montgomery County.

^f^SZ: Shenzhen.

^g^LBP: local binary pattern.

^h^SVM: support vector machine.

^i^We also evaluated the performance of these models (for the cross data set) using our selected data sets and experimental protocol.

^j^HoG: histogram of oriented gradients.

^k^—: not available. The results were not provided in this comparative study for these performance metrics.

## Discussion

**Figure 7:**
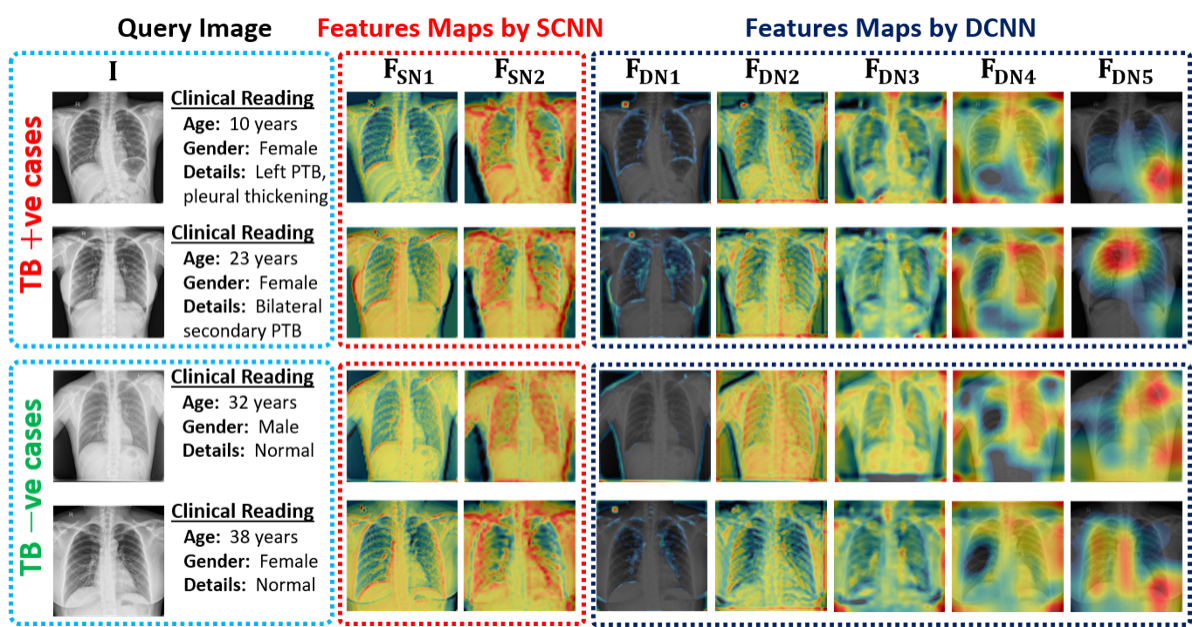
Extracted features maps from the different parts of the proposed ensemble-SDCNN model for both TB positive and negative cases. DCNN: deep convolutional neural network; SDCNN: shallow–deep convolutional neural network; SCNN: shallow convolutional neural network; TB: tuberculosis.

This article presents an interactive CAD framework based on multiscale information fusion to diagnose TB in CXR images and retrieve the relevant cases (CXR images) from a previous patients’ database including clinical observations. In this framework, a classification model is primarily proposed to classify the given CXR image as either a positive or a negative sample. Subsequently, classification-based retrieval is performed to retrieve the relevant cases and corresponding clinical readings based on our newly proposed MLSM algorithm. The proposed model substantially improves diagnostic performance by performing the fusion of both low- and high-level features. The network processes the input image through different layers and finally activates the class-specific discriminative region [48] as key-features maps. Figure 7 shows such activation maps extracted from the 7 different layers (ie, F_SN1_, F_SN2_, F_DN1_, F_DN2_, F_DN3_, F_DN4_, and F_DN5_ as labeled in Figure 2) of our model for both positive and negative sample images. As Figure 7 shows, each activation map is generated by calculating the average of all the extracted maps from a specific location. All the activation maps overlay on their corresponding input image after resizing and applying a pseudo-color scheme (blue to red, equivalent to lower to higher activated region) to produce a better visualization of the activated regions.

Figure 7 indicates that the class-specific discriminative regions of the given input image become more prominent after processing through the successive layers of the network. A semilocalized activation map (labeled as F_DN5_ in Figure 7) is obtained from the last convolutional layer of the DCNN model, which includes the more distinctive high-level features for each class. Moreover, for the SCNN, the obtained activation map from the last convolutional layer (labeled as F_SN2_ in Figure 7) encompasses the low-level features such as edge information. Finally, both low- and high-level features are used in making an effective diagnostic decision for the given CXR image. The experimental results (also provided in Multimedia Appendix 2) proved that the diagnostic performance of our ensemble-SDCNN model is more effective than the various CNN models where only single-level features are used for class prediction.

**Figure 8:**
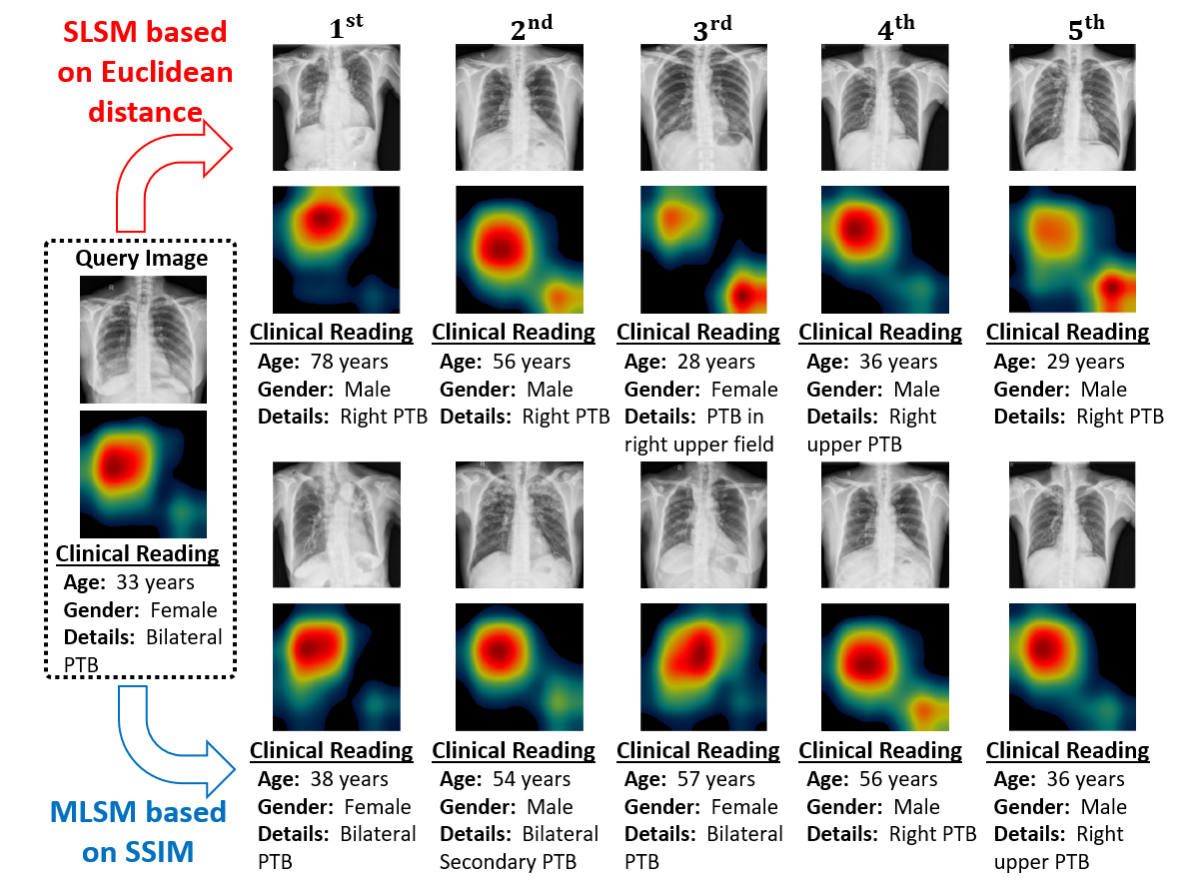
Visualization of retrieval performance for the given input query image by considering SLSM and MLSM (our proposed model). MLSM: multilevel similarity measure; SLSM: single-level similarity measure.

After an effective diagnostic decision, we can further retrieve the relevant cases based on our proposed MLSM algorithm, which considers the multilevel features in retrieving the best matches. Figure 8 depicts the retrieval results of our proposed MLSM algorithm in comparison with the conventional Euclidean distance–based SLSM scheme. In Figure 8, these results comprise the 5 best-matched CXR images along with their corresponding high-level activation maps (labeled as F_DN5_ in Figure 7) and clinical readings. Generally, a high correlation between the high-level activation maps (as F_DN5_ in our study) of the query image and retrieved image implies the optimal performance of a retrieval system. With our MLSM algorithm, these activation maps (corresponding to retrieved cases) were more analogous (in terms of shape and location) to that of query image compared with the conventional SLSM scheme. This implied that our algorithm retrieved the highly correlated cases in terms of TB patterns, location, and clinical observation.

**Figure 9:**
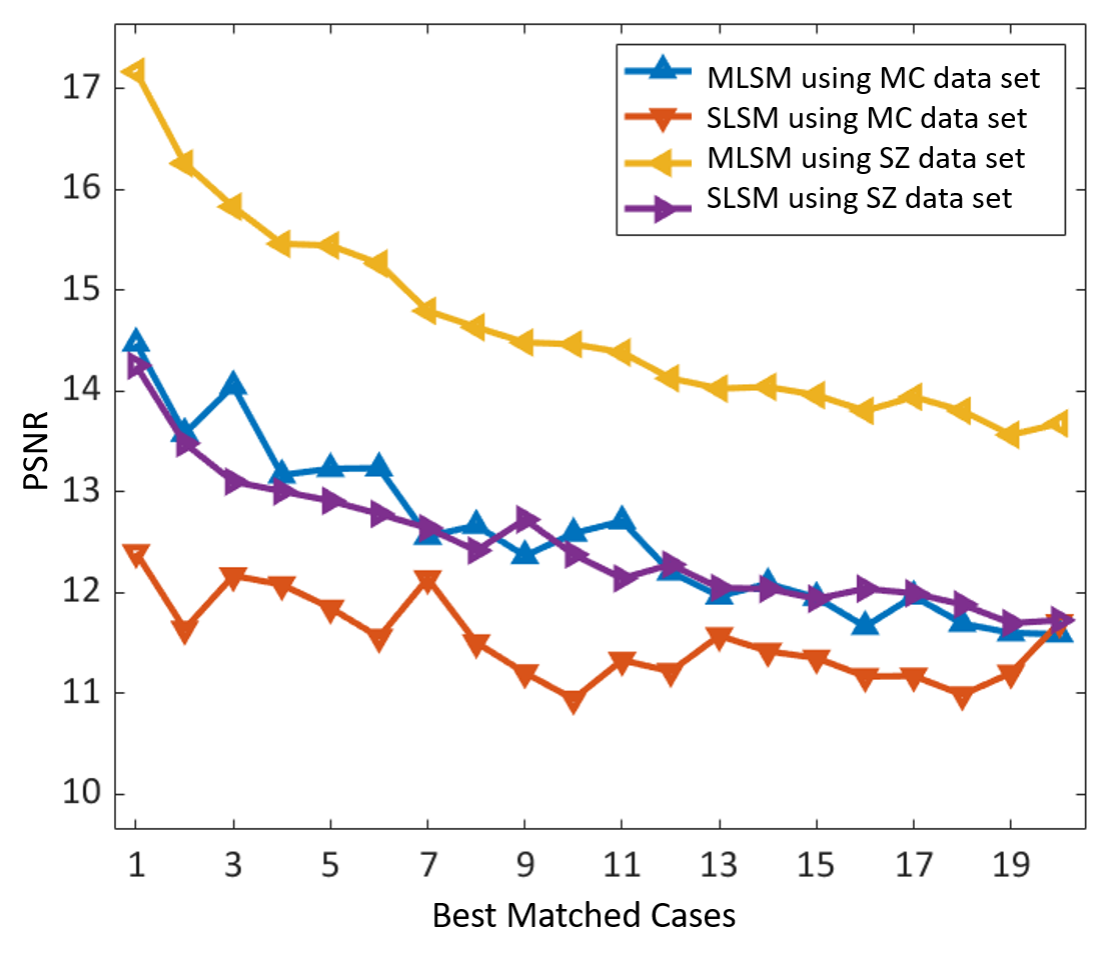
PSNR-based objective similarity measures between the high-level activation maps of the query image and retrieved images to evaluate feature-level similarities of both algorithms (ie, MLSM and SLSM). MLSM: multilevel similarity measure; PSNR: peak signal-to-noise ratio; SLSM: single-level similarity measure.

In addition, we evaluated the objective similarity score in terms of the PSNR between the activation maps of the input query and 20 best-matched cases for both algorithms (MLSM and SLSM). The main purpose of this analysis was to quantitatively evaluate such feature-level similarities of both algorithms. A total of 28 images (28/138, 20.2% of the MC data set) from the MC data set and 132 images (132/662, 19.9% of the SZ data set) from the SZ data set were selected as the query database to perform this analysis. Using each query image one at a time, we retrieved the 20 best-matched cases corresponding to each algorithm. Thus, 20 different PSNR values were computed corresponding to these retrieved images for each matching algorithm. After these results for the entire selected query database were evaluated, an average PSNR performance was calculated to present the average performance of a single query image for each algorithm. Figure 9 shows the comparative performance results of our proposed MLSM algorithm and the conventional SLSM scheme. We observed that our matching algorithm exhibited the higher features-level similarity scores in terms of the PSNR (for all the retrieved images and both data sets) in contrast to the SLSM scheme. Thus, our algorithm resulted in an optimal retrieval performance because of the significant correlation of high-level activation maps. All these results (Figures 8 and 9) were computed based on our selected classification-driven retrieval method. The experimental results provided in Table 4 have already proved that our selected class prediction–based retrieval method outperforms the retrieval method without class prediction.

**Figure 10:**
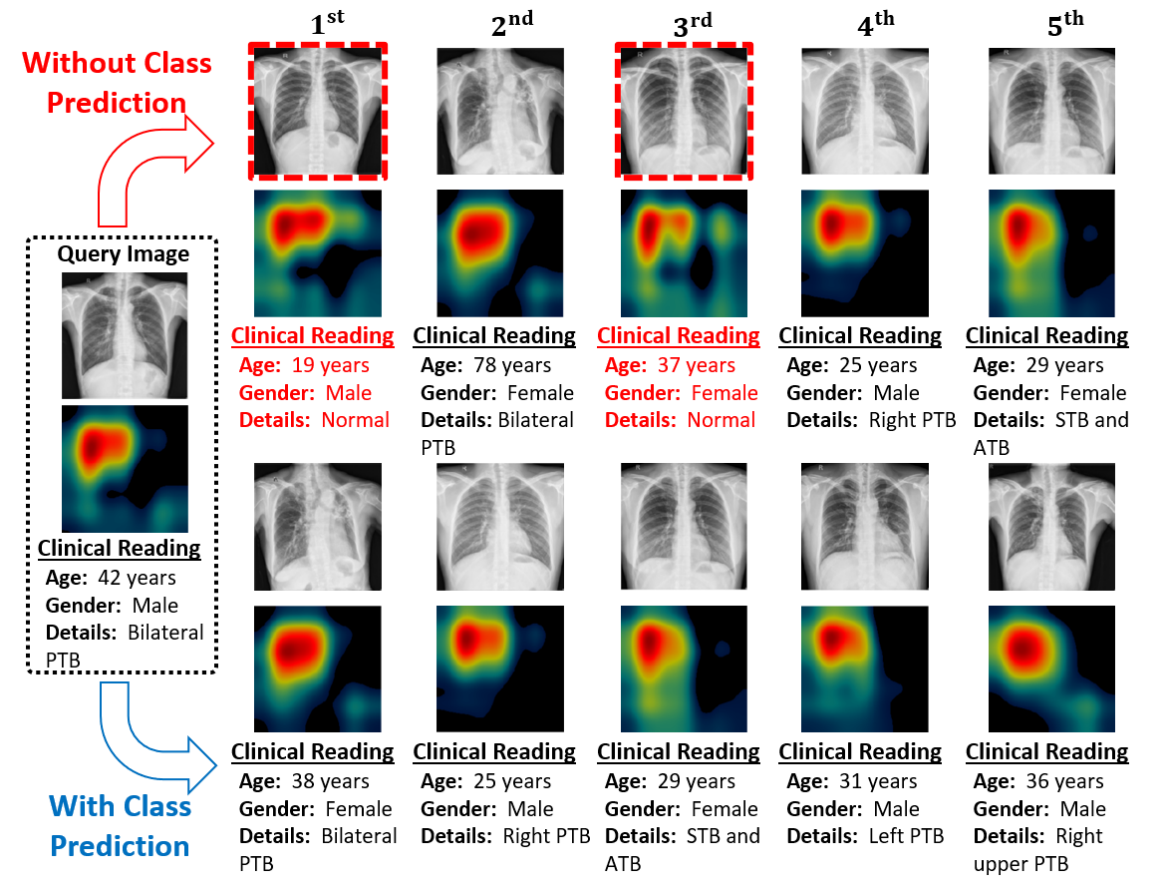
Visualization of retrieval performance for the given input query image by considering both retrieval methods with class prediction and without class prediction.

In addition to the numerical results provided in Table 4, Figure 10 further distinguishes the retrieved results of these 2 different approaches (ie, with and without class prediction) figuratively. Figure 10 indicates that all the retrieved cases (for the given query image) were TPs in our class prediction–based retrieval method.

However, in the retrieval without class prediction, the first and third best matches were FPs (highlighted by the red bounding box) while the remaining three cases were TPs. Such FP cases may lead to a vague diagnostic decision. Additionally, the numerical results (Table 4) indicated that the average number of FPs in retrieval without class prediction was substantially higher than our class-prediction retrieval method. Therefore, in this study, we considered a classification-driven retrieval by performing the class prediction in the first step and then retrieving the best-matched cases from the predicted class database rather than exploring the entire database. Ultimately, the classification results can aid in making a diagnostic decision and the retrieved CXR images can assist radiologists to further validate the computer decision. Furthermore, if the wrong prediction is made by the computer, the medical expert can check other relevant cases (ie, second-, third-, or fourth-best matches) that can be more relevant than the first best match. Thus, both classification and retrieval results can aid radiologists in making an effective diagnostic decision even in scenarios of small TB patterns that remain undetectable in the early stage. Such a comprehensive CAD framework may assist radiologists in clinical practices and alleviate the burden of an increasing number of patients by providing an effective and timely diagnostic decision. Our trained model and the training and testing data splitting information are publicly available [49] to enable other researchers to evaluate and compare its performance.

## Appendix

Multimedia Appendix 1 Other supplementary material is provided in the attached word file [DOCX file (MS Word), 44 KB].

Multimedia Appendix 2 All the experimental results are provided in the attached excel file [XLSX file (MS Excel), 226 KB].

## Abbreviations

ACC: accuracy
AP: average precision
AR: average recall
AUC: area under the curve
CAD: computer-aided diagnosis
CL: class label
CNN: convolutional neural network
CXR: chest radiograph
DCNN: deep convolutional neural network
FN: false negatives
FP: false positives
FPR: false-positive rate
F1: F1 score
HoG: histogram of oriented gradients
LBP: local binary pattern
MC: Montgomery County
MLSM: multilevel similarity measure
PSNR: peak signal-to-noise ratio
ROC: receiver operating characteristic (curve)
SDCNN: shallow–deep convolutional neural network
SCNN: shallow convolutional neural network
SLSM: single-level similarity measure
SSIM: structure similarity
SVM: support vector machine.
SZ: Shenzhen
TB: tuberculosis
TN: true negative
TP: true positive
TPR: true-positive rate
WHO: World Health Organization
