# Supplementary material for: Comprehensive Computer-Aided Decision Support Framework to Diagnose Tuberculosis From Chest X-Ray Images: Data Mining Study
Source: JMIR Med Inform. 2020 Dec 7;8(12):e21790. doi: 10.2196/21790 (PMC7752539; doi:10.2196/21790)
Supplement: Multimedia Appendix 1 [file medinform_v8i12e21790_app1.docx]

## **Multimedia Appendix 1**

**Authors**

Muhammad Owais, MSc; Muhammad Arsalan, PhD; Tahir Mahmood, MSc; Yu Hwan Kim, MSc; Kang Ryoung Park, PhD

**Affiliations:**

Division of Electronics and Electrical Engineering, Dongguk University, Seoul, Korea

Emails: [malikowais266@gmail.com](mailto:malikowais266@gmail.com) (M.O); [arsal@dongguk.edu](mailto:arsal@dongguk.edu) (M. A.); [tahirmahmood@dongguk.edu](mailto:tahirmahmood@dongguk.edu) (T.M.); [taekkuon@naver.com](mailto:taekkuon@naver.com) (Y.H.K.)

**Corresponding Author:**

Kang Ryoung Park, PhD

Division of Electronics and Electrical Engineering

Dongguk University

30 Pildong-ro 1-gil, Jung-gu

Seoul, 04620

Republic of Korea

Phone: 82 10 3111 7022

Fax: 82 2 2277 8735

Email: [parkgr@dgu.edu](mailto:parkgr@dgu.edu)

## **Related Work**

The evolutional era of CAD tools in the field of chest radiography can be divided into two parts based on the selected set of algorithms for their development. The early development primarily consisted of conventional hand-crafted feature-based algorithms. Later, the set of deep learning algorithms replaced such hand-crafted methods by exhibiting a remarkable performance gain of CAD tools in chest radiography. In this related work section, we summarize the recent literature in the development of CAD tools based on both hand-crafted and deep learning algorithms.

In recent decades, several ground-breaking CAD methods have been proposed for TB diagnosis [9-24]. Most previous studies used the segmentation, detection, and classification-based approaches to make ultimate diagnostic decisions. For example, Jaeger et al. [9] proposed a method involving segmentation followed by classification for the early screening of TB using a CXR image. In that study, a graph-cut method was used to segment the lungs region; subsequently, a set of low-level features (such as shape and texture features) was extracted to classify the CXR image either as normal or abnormal using a binary classifier. Later, Kumar et al. [10] presented a classification approach to distinguish between normal lungs and pulmonary edema in the CXR image by using a Gabor filter and support vector machine (SVM). In another study by Hogeweg et al. [11], a similar approach as presented in [9] was extended by combining focal information with texture and shape features. The combination of all these features (i.e., focal, texture, and shape) incorporated the diverse patterns related to disease, which ultimately resulted in better performance.

Another well-known descriptor of texture features, local binary patterns (LBP), was used to extract TB patterns from segmented CXR images in [12]. The segmented lungs region was extracted using a template matching algorithm. However, the overall pipeline of their method was also similar to that by [9,11]. Similarly, in [13], Karargyris et al. extracted shape and texture features to detect the pulmonary abnormalities in CXR images. A novel Atlas-based lung segmentation approach was presented to extract the shape features; different features descriptors (intensity histogram, gradient magnitude histograms, histogram of oriented gradients (HoG), and LBP) were then applied over a lung region of interest (ROI) to extract the texture information. Finally, the SVM classifier was used to classify the given CXR image based on these multilevel shape and texture features. However, the computational complexity of such multi-level feature descriptors is significantly high compared with a single-level feature descriptor. To reduce the computation complexity of a CAD tool, Alfadhli et al. [14] used a single Speeded-up robust features (SURF) descriptor in diagnosing TB. As SURF is a local feature descriptor, it required less computational power than other multi-level features descriptors used in [11,13]. Later, Govindarajan et al. [15] combined a bag-of-features (BoF) method with a SURF keypoint descriptor to further enhance the classification performance using a multilayer perceptron (MLP) classifier. In a subjective radiological examination, lung region symmetry information is used to diagnose TB by performing the bilateral comparison of the lung field. In a recent study, a novel hand-crafted features descriptor was proposed [16] to classify the pulmonary abnormalities in a fully automated manner. In that study, symmetry information was considered with shape and texture features to classify the abnormal CXR images using an ensemble classifier.

In addition to these conventional hand-crafted feature-based methods, some recent papers [17-24] utilized the strength of deep learning algorithms to detect and precisely classify the different types of pulmonary abnormalities. In this new paradigm, a network is trained to extract discriminative features from given CXR images in a fully automated manner. For example, Hwang et al. [17] proposed a fully automated CNN-based CAD tool to perform TB diagnosis. In that study, a shallow CNN model was designed to include a total of six convolutional layers and three fully connected (FC) layers. Subsequently, a transfer learning strategy was adopted to perform the end-to-end training of the network. In addition to these binary diagnostic tools (i.e., either TB positive or TB negative), Shin et al. [18] proposed a CNN and a classification model based on a recurrent neural network (RNN) to diagnose the different types of chest abnormalities. In this classification problem, they considered a total of thirteen different classes related to chest abnormalities and included a normal class. However, the overall diagnostic performance of their proposed model was not sufficient (accuracy of 69.84% for a validation dataset) to make it suitable in clinical practices.

Later, Lakhani et al. [19] proposed an ensemble of two well-known CNN models (AlexNet [25] and GoogLeNet [20]) to classify pulmonary TB cases either as positive or negative. They also considered a subjective evaluation scheme for some cases where two CNN models were in disagreement. Thus, they further decreased the number of false positives. In another study [21], the authors proposed three different proposals for TB diagnosis using three pre-trained CNN models. In the first proposal, the extracted features from these CNN models were classified by training an SVM classifier. In the second proposal, the same CNN models were used to extract features from subregions of the CXR images. Subsequently, all these features were combined and classified using an SVM classifier. In the final proposal, an ensemble of the best SVM classifiers (trained on proposal 1 and proposal 2) was created to further enhance the overall performance. The majority of previous studies utilize existing CNN models (proposed for general image classification) in TB diagnostic scenarios. In a recent study, Pasa et al. [22] proposed an optimized CNN model for the effective screening and visualization of TB. The number of trainable parameters of their proposed model was significantly lower than those of various pre-trained CNN models. However, we observed that its performance was lower than that of the various pre-trained CNN models. In the medical domain, the detection performance of any CAD tool is more crucial than its computational cost.

In most recent studies, Qin et al. [23] and Nash et al. [24] presented a retrospective case-control study to assess the diagnostic performance of existing CAD software for detecting TB-associated abnormalities in CXR images. In both studies, different CXR datasets were collected from hospitals, and a detailed performance analysis was performed. Table 1 provides a summary of these existing methods along with our proposed framework. Although in the last few years many papers have been published on the automated diagnosis of TB using CXR images, a performance gap remains between these proposed methods and actual deployable CAD systems. All the above mentioned studies [9-24] indicated a binary decision (either TB positive or TB negative) without providing further descriptive information that may assist medical experts to validate the CAD decision. As the CAD decision can also be erroneous in some scenarios, a method to perform its cross-validation is necessary. Therefore, further research is required to achieve the practical performance and usability of such diagnostic systems in the real world.

**Table 1.** Comparative summary of our proposed and existing state-of-the-art methods in TB diagnostic Domain. This table highlights the key differences of various existing methods in comparison with our proposed method along with their strengths and weaknesses.

| **Methodology** | | **Cross-dataset** | **Strength** | **Weakness** |
| --- | --- | --- | --- | --- |
| **Hand-crafted features** | Graph cut, shape, and texture features + SVM [9] | No | Scale-invariant features | Low classification accuracy |
|  | Manual segmentation, Gabor filter + SVM [10] | No | Computationally efficient | Limited dataset (80 images) |
|  | Focal, texture, and shape features [11] | No | CAD performance close to radiologists | Moderate AUC performance |
|  | Template matching, LBP [12] | No | Detection rate is high | Limited dataset (48 images) |
|  | Low-level texture features + SVM [13] | No | Overall AUC is high | Limited performance comparison |
|  | SURF + SVM [14] | No | Computationally efficient | Limited dataset (138 images) |
|  | BoF, SURF + MLP classifier [15] | No | Computationally efficient | Limited dataset (138 images) |
|  | Shape and textures features + ensemble classifier [16] | Yes | Scale and rotation invariant features | Low classification accuracy |
| **Deep features** | Deep features using shallow CNN [17] | Yes | Large dataset and computational efficient | Limited performance comparison |
|  | Deep CNN and RNN [18] | No | Large dataset with high training accuracy | Low validation accuracy |
|  | An ensemble of two CNNs [19] | No | High sensitivity and specificity | Limited performance analysis |
|  | Bag of CNN features + SVM [21] | No | Overall AUC is high | Limited performance comparison |
|  | Optimized shallow CNN [22] | No | Computational efficient | Overall accuracy is limited |
|  | CNN-based CAD tools [23] | No | Higher performance than radiologists | Low sensitivity |
|  | CNN-based CAD tools [24] | No | Higher performance than radiologists | Moderate sensitivity and specificity |
|  | **Proposed** | Yes | Enhanced performance and detailed analysis | Higher retrieval time than classification |
